# Idiopathic Gingival Hyperplasia: A Case Report with a 17-Year Followup

**DOI:** 10.1155/2011/986237

**Published:** 2011-07-03

**Authors:** Bien Lai, Joseph Muenzer, Michael W. Roberts

**Affiliations:** ^1^Department of Pediatric Dentistry, CB 7450, School of Dentistry, University of North Carolina, Chapel Hill, NC 27599-7450, USA; ^2^Division of Pediatric Genetics and Metabolism, Department of Pediatrics, CB 7484, School of Medicine, University of North Carolina, Chapel Hill, NC, USA

## Abstract

This is a case report of a patient with idiopathic gingival hyperplasia and an undiagnosed genetic disorder that demonstrated static encephalopathy, mental retardation, developmental delay, seizures, hypotonia, and severe gingival hypertrophy. The clinical dental management and attempts to obtain a genetic diagnosis are described.

## 1. Introduction

Gingival hyperplasia can occur as an isolated form or part of a syndrome. Complications associated with gingival overgrowth may include retained primary teeth, delayed eruption of permanent teeth, increased distal spacing, drifting of teeth, poor plaque control, poor mastication, affected speech, esthetics, and malocclusion [1–4]. Several etiologies have been reported including drug-induced, hereditary, hormones-related (pregnancy, growth-hormone), inflammation, systemic (leukemia, neurofibromatosis), idiopathic, and syndrome associated. Depending on the cause, the overgrowth may vary in clinical presentation, severity, onset, and duration.

Cyclosporine, phenytoin, and nifedipine are the most common drugs associated with drug-induced gingival overgrowth [4–10]. The affect is more commonly observed in children than adults, possibly due to immature fibroblasts having increased sensitivity to cyclosporine [8, 9]. Synergistic effects of nifedipine and cyclosporine have also been reported [11, 12].

Rare cases of sodium valproate-related gingival enlargement have also been reported, including one involving infantile gingival overgrowth at birth secondary to maternal sodium valproate use [13–16]. However, a clinical study involving epileptic adults receiving sodium valproate and a control group showed no significant differences between the two groups on any parameters assessed [6]. 

Hereditary gingival hyperplasia is a slowly progressive, generalized, severe gingival enlargement involving maxillary and mandibular arches. The pink firm gingiva is fibrotic and nonhemorrhagic. Severity varies and may cover part/all of the crowns of the erupted teeth. It usually develops around the eruption of permanent teeth and is rarely present at birth. The most common mode of inheritance is autosomal dominant with variable penetrance [17–19]. The syndrome most commonly associated with gingival hyperplasia is gingival fibromatosis with generalized hypertrichosis, mental retardation, and tonic-clonic seizures [20, 21]. Isolated cases of gingival fibromatosis associated with amelogenesis imperfect and aggressive periodontitis have also been reported [2, 22, 23]. Conditions known to be associated with gingival hyperplasia are summarized in Table 1.

Idiopathic gingival hyperplasia has been described in several case reports. The clinical presentation of the gingiva was similar to that of hereditary gingival hyperplasia but did not have a contributory medical or family history [1–3]. General histological findings include normal overlying epithelium, rete pegs extending deep into underlying connective tissues with some areas of hyperplasia, proliferating dense fibrous CT with increased cellularity and coarse collagenous fiber bundles and hyperkeratosis and acanthosis with elongated papillae [1, 2, 18].

The purpose of this paper is to present a case report of a patient with idiopathic gingival hyperplasia and an undiagnosed medical condition that demonstrated static encephalopathy, mental retardation, developmental delay, seizures, and hypotonia. 

## 2. Case Report

A 2-month old Caucasian male was admitted to the hospital due to right perihilar pneumonia with hyperaeration and three months later was diagnosed with chicken pox. He also presented with global delays, dysmorphic features including craniofacial asymmetry, apparently secondary to postural position, with flattening of the left occiput, cup-shaped external ears, broad nasal bridging and small upturned tip of the nose, smooth philtrum, umbilical hernia, undescended testes, lax abdominal muscles, overlapping toes, and broad and thickened hands and feet. He was subsequently referred for genetic and neurologic evaluation. Peripheral blood taken for chromosomal analysis revealed no abnormalities, including negative results for Fragile X syndrome. A computed tomography (CT) scan of the head revealed atrophic changes of the brain, with no hemorrhage or evidence of masses or recent infarction. 

The child's mother reported an uncomplicated pregnancy and denied prenatal exposure to tobacco, alcohol, and drugs. The only medication taken during pregnancy was a suppository for nausea. She also reported no exposure to radiation and no illness during her pregnancy. The birth history was unremarkable, with 40-week gestation and normal spontaneous vaginal delivery. Prenatal history was uneventful with no infection, bleeding, or unusual event. The patient exhibited no jaundice or cyanosis at birth. There was no information about newborn screening for PKU, hypothyroidism, or galactosemia. No history to suggest consanguinity was reported by the parents and there was no family history to suggest birth defects or mental retardation. The affected child had a healthy 2.5-year-old female sibling.

The patient suffered from repeated respiratory infection over the next 4-5 months and had an adenoidectomy at ten months of age. He continued to demonstrate developmental delays, spasticity of the upper and lower extremities, and poor muscle tone. A neurology evaluation reported that the growth and developmental course did not support a progressive or degenerative condition. It was suggested that the overall developmental delay was the result of some unknown intrauterine encephalopathy or developmental anomaly of the brain.

At 21 months of age the patient was seen in the pediatric clinic for well child care. It was reported that he had been seen by a dentist and there was no evidence of dental eruption. He was prescribed with theophylline, albuterol sulfate, and Naldecon for his asthma-like symptoms and recurrent respiratory infections. The patient was referred at 24 months of age to an occupational therapist to assist with mouth motions and lip movements. He was reported to have severe generalized hypotonia and hypertrophic gums with only four partially erupted mandibular incisors. 

**Figure 1:**
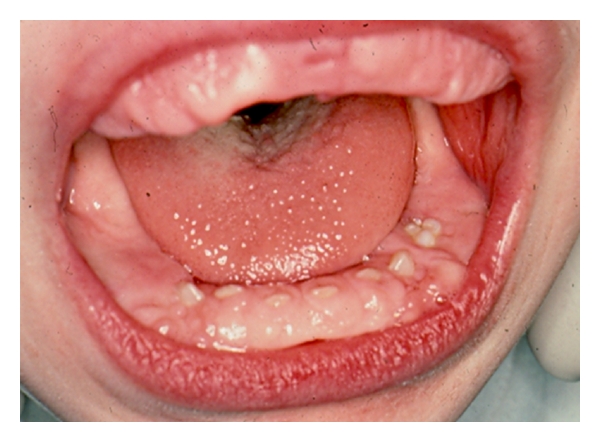
Pre-operative picture of gingival hyperplasia at 4 years/6 months of age.

**Figure 2:**
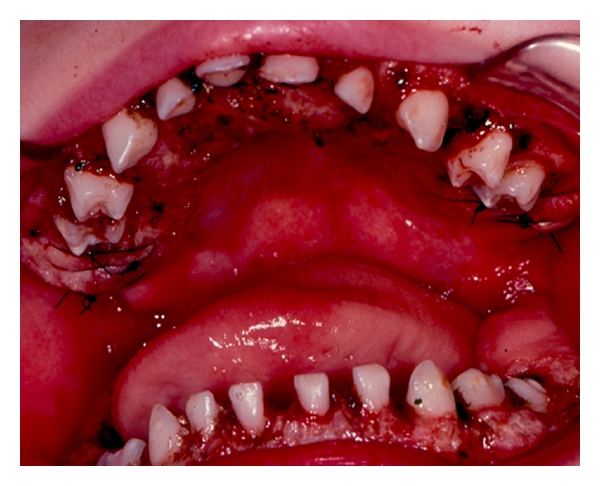
Immediate post-gingivectomy.

**Figure 3:**
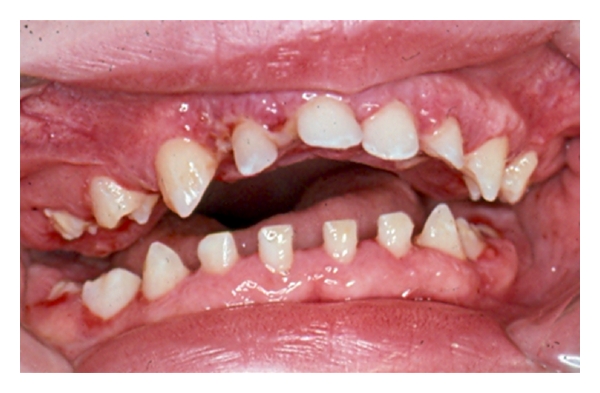
10-day after surgery.

Four months later, the patient was seen at a developmental evaluation center. Findings included diffuse encephalopathy of unknown origin, dysmorphic features, microcephaly, generalized hypotonia, and oral-motor dysfunction. There were multidisciplinary follow-up visits with an ophthalmologist, urologist, dentist, audiologist, speech, and language therapist. The patient was referred to the Department of Pediatric Dentistry, University of North Carolina School of Dentistry for evaluation and management of gingival hyperplasia. At 4.5 years of age, a full mouth gingivectomy was completed using a carbon dioxide laser and electrosurgery, under general anesthesia. The patient tolerated the procedure well and was discharged the following day (Figures 1 and 2). He was seen 10 days after-surgery for evaluation. The postoperative course was uneventful. Oral hygiene was emphasized to parents (Figure 3).

**Figure 4:**
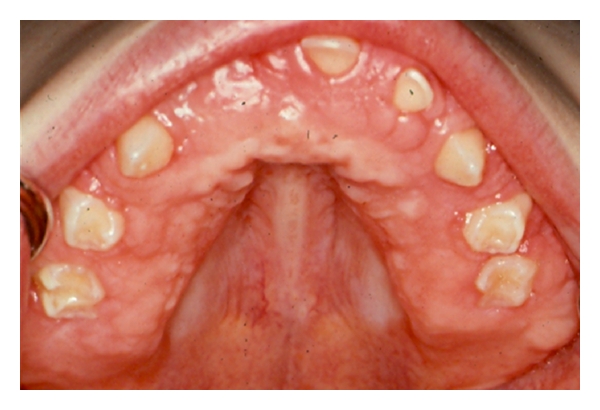
Recurrence of gingival hyperplasia at 6 years of age (primary dentition).

When examined 16 months later (6 years of age), the gingival hyperplasia had recurred for on both maxillary and mandibular dental arches (Figure 4). The parent provided oral hygiene for the patient was excellent. The patient was taking divalproex sodium for his seizure disorder. Again, a full mouth gingivectomy and extraction of some primary anterior teeth that were near exfoliation was provided under general anesthesia. The primary teeth and multiple gingiva specimens were sent to pathology. Dense fibrous connective gingiva tissue with no Periodic acid-Schiff (PAS) stainable material in the specimen was reported. A pediatric geneticist in the Division of Pediatric Genetics and Metabolism recommended various tests for possible rare storage disorders. Chromosomal testing, mucopolysaccharides (MPS), and lysosomal disorder screenings were negative. Peritoneal and skin biopsies were obtained and sent to cytogenetics for investigation. Results of the various tests revealed no abnormalities and no definitive diagnosis were made.

Over the next three years, the patient was subjected to his third and fourth gingivectomy procedures under general anesthesia due to recurrence of gingival hyperplasia. At 11 years 4 months of age, he was subjected to a fifth gingivectomy procedure. He was still taking divalproex sodium for his seizure disorder.

The patient presented for another dental examination at the age of 16 years. He continued to demonstrate gingival hyperplasia. A consult from the Department of Oral and Maxillofacial Surgery was obtained. The suggested treatment was extraction of all teeth in attempt to reduce the current gingival hyperplasia, remove excess alveolar bone, and possibly prevent future gingival overgrowth versus no treatment. There was no subsequent followup by the parents.

**Figure 5:**
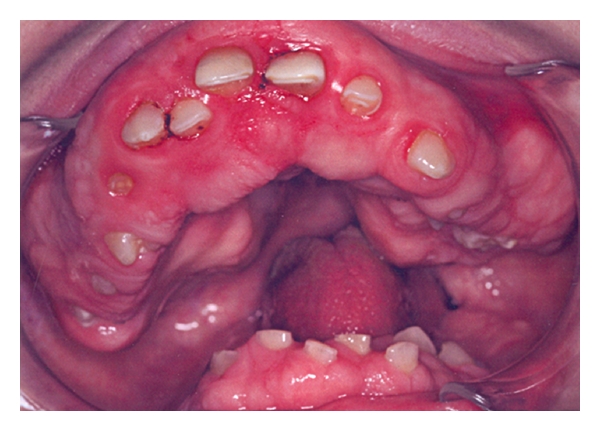
Recurrence of gingival hyperplasia at 16 years of age (permanent dentition).

Four years later (20 years of age), the patient and his parents returned for another dental evaluation with regard to the continued gingival hyperplasia (Figure 5). His open bite and lip incompetence had increased from the last dental visit. The oral surgeon from the Department of Oral and Maxillofacial Surgery was again consulted who suggested that total odontectomy and alveoplasty was the treatment of choice. The parents agreed but the family had moved to another state and chose to seek definitive dental care closer to their home. 

## 3. Discussion

Gingival hyperplasia is thought to be caused by one or more sources. These include an increase in proliferation of resident tissue fibroblasts, a reduced level of metalloproteinases synthesis (MMP-1 and MMP-2), resulting in low levels of extracellular matrix-degrading, an increase in collagen type I production, heat-shock protein 47 (hsp47) production, and other extracellular matrix components [19].

Gagliano et al. [3] suggested that gingival hyperplasia of different etiologies may have different mechanisms of overgrowth. The likely mechanism of idiopathic gingival hyperplasia may be increased collagen deposition as a consequence of post translational mechanisms. The increase in collagen cross-links renders it less susceptibility to MMP degradation, favoring its accumulation in the gingival connective compartment. Cyclosporine-induced gingival hyperplasia is associated with a decrease in the breakdown of interstitial collagen with a low level of MMP, while hereditary gingival fibromatosis demonstrates an activated fibroblasts phenotype. 

In this case report, the patient was presented with static encephalopathy of unknown origin, dysmorphic features, microcephaly, generalized hypotonia, and seizure disorder. Multiple tests were completed in an attempt to diagnose his condition, but no genetic abnormalities were detected. The patient also had delayed eruption of his primary teeth, with no primary teeth present at 21 months old, and only four mandibular incisors at 24 months of age. Gingival hyperplasia of unknown etiology was first noted at 24 months of age and before he was placed on divalproex sodium for seizure disorder. This gingiva growth continued to recur throughout the years, resulting in increased lip incompetence and overbite. The etiology of the patient's gingival hyperplasia remains unknown. With no family history and no apparent association with any medical syndromes, the impression is that the gingival hyperplasia is idiopathic in nature and possibly exacerbated by some drug influence from divalproex sodium, which has been reported to have controversial effects on gingival tissues [6, 13–16]. It could also be a rare mutation or other hereditary disorder with some variance in expression such that other family members are not affected. However, this could not be confirmed as no genetic testing was completed on the family members.

Management of gingival hyperplasia depends on the cause of the condition. Drug-induced gingival hyperplasia may improve with substitution of other drugs that rarely affect the gingiva, such as barbiturates, valproic acid, and tacrolimus [6, 24–29]. Use of antibiotics such as metronidazole and azithromycin for cyclosporine-induced gingival hyperplasia has been reported [30–32]. In general, reinforcement of good home care oral hygiene regimens and periodic professional surgical excision of gingival are the treatments of choice [1–3, 19, 33].

The present case describes a patient with an undiagnosed medical condition associated with idiopathic gingival hyperplasia and its management. It demonstrates the complex nature of many genetic disorders that have not yet been described but present treatment challenges. 

## Figures and Tables

**Table 1:** 

Conditions associated with gingival hyperplasia	Features
Autosomal recessive	
I-cell disease (Mucolipidosis II)	Mental and physical retardation; appears prior to eruption of primary teeth
Ramon Syndrome	Gingival fibromatosis, hypertrichosis, cherubism, mental retardation, and seizures
Juvenile hyaline fibromatosis (Murray-Peretic-Drescher syndrome)	Multiple hyaline fibromas, white papules on the skin, flexion contractures, osteolytic bone lesions, and gingival fibromatosis
Alpha-Mannosidosis	A type of oligosaccharidosis, delayed early motor development, mild hypotonia, hypoplastic bones, macroglossia, hepatosplenomegaly, and gingival enlargement
Donohue syndrome (Leprechaunism)	Failure to thrive, unusual facies, facial hirsutism, retarded bone age, and insulin resistance with glucose intolerance and hyperinsulinemia
Cross syndrome	Hypopigmentation, microphthalmia, mental retardation, athetosis, and gingival fibromatosis
Hornova-Dluhosova syndrome	Oral and conjunctival amyloidosis and mental retardation

Autosomal dominant	
Zimmerman-Laband syndrome	Gingival fibromatosis, ear, bone, nail defects, hepatosplenomegaly
Rutherford syndrome	Gingival fibromatosis and corneal dystrophy, failure of tooth eruption
Jones syndrome	Gingival fibromatosis with sensorineural hearing loss

Other	
Borronedermato-cardio-skeletal syndrom (Autosomal recessive/X-linked recessive)	Coarse facies, thick skin, acne conglobata, gingival enlargement, osteolysis, camptodactyly, and mitral valve prolapsed

## References

[1] Kavvadia K, Pepelassi E, Alexandridis C, Arkadopoulou A, Polyzois G, Tossios K (2005). Gingival fibromatosis and significant tooth eruption delay in an 11-year-old male: a 30-month follow-up. *International Journal of Paediatric Dentistry*.

[2] Chaturvedi R (2009). Idiopathic gingival fibromatosis associated with generalized aggressive periodontitis: a case report. *Journal of the Canadian Dental Association*.

[3] Gagliano N, Moscheni C, Dellavia C (2005). Morphological and molecular analysis of idiopathic gingival fibromatosis: a case report. *Journal of Clinical Periodontology*.

[4] Doufexi A, Mina M, Ioannidou E (2005). Gingival overgrowth in children: epidemiology, pathogenesis, and complications. A literature review. *Journal of Periodontology*.

[5] Wright G, Welbury RR, Hosey MT (2005). Cyclosporin-induced gingival overgrowth in children. *International Journal of Paediatric Dentistry*.

[6] Seymour RA, Smith DG, Turnbull DN (1985). The effects of phenytoin and sodium valproate on the periodontal health of adult epileptic patients. *Journal of Clinical Periodontology*.

[7] Herranz JL, Armijo JA, Arteaga R (1988). Clinical side effects of phenobarbital, primidone, phenytoin, carbamazepine, and valproate during monotherapy in children. *Epilepsia*.

[8] Leppik IE (1992). Metabolism of antiepileptic medication: newborn to elderly. *Epilepsia*.

[9] Chabria D, Weintraub RG, Kilpatrick NM (2003). Mechanisms and management of gingival overgrowth in paediatric transplant recipients: a review. *International Journal of Paediatric Dentistry*.

[10] Barclay S, Thomason JM, Idle JR, Seymour RA (1992). The incidence and severity of nifedipine-induced gingival overgrowth. *Journal of Clinical Periodontology*.

[11] Thomason JM, Seymour RA, Rice N (1993). The prevalence and severity of cyclosporin and nifedipine-induced gingival overgrowth. *Journal of Clinical Periodontology*.

[12] O'Valle F, Mesa F, Aneiros J (1995). Gingival overgrowth induced by nifedipine and cyclosporin A. Clinical and morphometric study with image analysis. *Journal of Clinical Periodontology*.

[13] Behari M (1991). Gingival hyperplasia due to sodium valproate. *Journal of Neurology Neurosurgery and Psychiatry*.

[14] Anderson HH, Rapley JW, Williams DR (1997). Gingival overgrowth with valproic acid: a case report. *Journal of Dentistry for Children*.

[15] Syrjänen SM, Syrjänen KJ (1979). Hyperplastic gingivitis in a child receiving sodium valproate treatment. *Proceedings of the Finnish Dental Society*.

[16] Rodríguez-Vázquez M, Carrascosa-Romero MC, Pardal-Fernández JM, Iniesta I (2007). Congenital gingival hyperplasia in a neonate with foetal valproate syndrome. *Neuropediatrics*.

[17] Hart TC, Pallos D, Bozzo L (2000). Evidence of genetic heterogeneity for hereditary gingival fibromatosis. *Journal of Dental Research*.

[18] Bozzo L, Machado MA, de Almeida OP, Lopes MA, Coletta RD (2000). Hereditary gingival fibromatosis: report of three cases. *Journal of Clinical Pediatric Dentistry*.

[19] Coletta RD, Graner E (2006). Hereditary gingival fibromatosis: a systematic review. *Journal of Periodontology*.

[20] Douzgou S, Mingarelli R, Dallapiccola B (2009). Gingival overgrowth, congenital generalized hypertrichosis, mental retardation and epilepsy: case report and overview. *Clinical Dysmorphology*.

[21] Gorlin RJ, Cohen MM, Levin LS (2001). Syndromes with Gingival/Periodontal components. *Syndromes of the Head and Neck*.

[22] Roquebert D, Champsaur A, del Real PG (2008). Amelogenesis imperfecta, rough hypoplastic type, dental follicular hamartomas and gingival hyperplasia: report of a case from Central America and review of the literature. *Oral Surgery, Oral Medicine, Oral Pathology, Oral Radiology and Endodontology*.

[23] Casavecchia P, Uzel MI, Kantarci A (2004). Hereditary gingival fibromatosis associated with generalized aggressive periodontitis: a case report. *Journal of Periodontology*.

[24] Lafzi A, Farahani RMZ, Shoja MAM (2007). Phenobarbital-induced gingival hyperplasia. *Journal of Contemporary Dental Practice*.

[25] Sinha S, Kamath V, Arunodaya GR, Taly AB (2002). Phenobarbitone induced gingival hyperplasia. *Journal of Neurology Neurosurgery and Psychiatry*.

[26] Reynolds NC, Kirkham DB (1980). Therapeutic alternatives in phenytoin-induced gingival hyperplasia. A case report and discussion. *Journal of Periodontology*.

[27] Shiboski CH, Krishnan S, Den Besten P (2009). Gingival enlargement in pediatric organ transplant recipients in relation to tacrolimus-based immunosuppressive regimens. *Pediatric Dentistry*.

[28] Greenberg KV, Armitage GC, Shiboski CH (2008). Gingival enlargement among renal transplant recipients in the era of new-generation immunosuppressants. *Journal of Periodontology*.

[29] Sekiguchi RT, Paixão CG, Saraiva L, Romito GA, Pannuti CM, Lotufo RFM (2007). Incidence of tacrolimus-induced gingival overgrowth in the absence of calcium channel blockers: a short-term study. *Journal of Clinical Periodontology*.

[30] Gómez E, Sánchez-Nuñez M, Sánchez JE (1997). Treatment of cyclosporin-induced gingival hyperplasia with azithromycin. *Nephrology Dialysis Transplantation*.

[31] Jucgla A, Moreso F, Sais G (1998). The use of azithromycin for cyclosporin-induced gingival overgrowth. *British Journal of Dermatology*.

[32] Wong W, Hodge MG, Lewis A, Sharpstone P, Kingswood JC (1994). Resolution of cyclosporin-induced gingival hypertrophy with metronidazole. *The Lancet*.

[33] Karpinia KA, Matt M, Fennell III RS, Hefti AF (1996). Factors affecting cyclosporine-induced gingival overgrowth in pediatric renal transplant recipients. *Pediatric Dentistry*.

